# Education and exercise program improves osteoporosis knowledge and changes calcium and vitamin D dietary intake in community dwelling elderly

**DOI:** 10.1186/s12889-017-4966-4

**Published:** 2017-12-19

**Authors:** Ki-Soo Park, Jun-Il Yoo, Ha-Young Kim, Sunmee Jang, Yongsoon Park, Yong-Chan Ha

**Affiliations:** 1 Department of Preventive Medicine and Institute of Health Sciences, Gyeongsang National University Hospital, Jinju, South Korea; 2 Department of Orthopaedic Surgery, School of Medicine, Gyeongsang National University, Jinju, Gyeongsangnam-Do South Korea; 3 Department of Internal Medicine, School of Medicine, Wonkwang University Gunpo Hospital, Gunpo, South Korea; 4 College of Pharmacy, Gachon University, Incheon, South Korea; 5 Department of Food and Nutrition, College of Human Ecology, Hanyang University, Seoul, South Korea; 6 Department of Orthopaedic Surgery, Chung-Ang University College of Medicine, 102 Heukseok-ro, Dongjak-ku, Seoul, 06973 South Korea

**Keywords:** Calcium, Education, Exercise, Osteoporosis knowledge, Vitamin D

## Abstract

**Background:**

Several educational intervention programs have been designed and developed to improve osteoporosis diagnosis and treatment. However, most of the prior studies focused on how educational intervention programs affected diagnosis and treatment of condition of osteoporosis.

The purpose of this prospective and educational intervention study was to evaluate the changes in osteoporosis knowledge, osteoporosis self-efficacy, fall self-efficacy, physical exercise and changes in dietary pattern of calcium and vitamin D intake after osteoporosis education.

**Methods:**

From November 1, 2015 to August 31, 2016, 271 eligible candidates (who were over 50 years old and from 23 different community centers) were recruited through an announcement made by the public office, by two health care providers.

The intervention involved an individualized education program to allow for differences in antecedent educational levels regarding several aspects of osteoporosis, including osteoporosis knowledge, osteoporosis self-efficacy, awareness of self-efficacy risk factors relating to an accidental fall and nutritional education (including the importance of sufficient calcium and vitamin D intake). The researchers revisited the community centers three months after the initial visit.

**Results:**

Of the 271 potential participants, 199 (73.4%; 43 men and 156 women) completed the education program and the second questionnaire. After education intervention, parameters including osteoporosis knowledge, osteoporosis self-efficacy and fall self-efficacy were improved (*P* < 0.0001). After education regarding percentage of calcium and vitamin D intake below recommended cut-offs, inadequate dietary calcium and vitamin D intake were decreased (*P* < 0.0001) from 89.4% (178/199) and 84.4% (168/199) to 79.9% (159/199) and 65.8% (131/199), respectively, at the three-month follow-up. (*p* = 0.038, *p* = 0.017).

**Conclusions:**

This prospective intervention study demonstrated that education on osteoporosis knowledge and regular exercise programs could improve osteoporosis self- efficacy, fall self-efficacy and increase dietary calcium and vitamin D intake.

## Background

Osteoporosis, a well-known age-related disease, is a pathology characterized by decreased bone density and regularly results in fractures, which in turn increase the morbidity rate in the elderly population. The pathology is associated with high mortality and socio-economic stress [1–3]. The prevalence of osteoporosis in the general population, aged 50 years or more in Korea, has been reported to be 37.3% in women and 7.5% in men [4]. Although osteoporosis is treatable and it is possible to sometimes obviate osteoporotic fractures, approximately 30% of women will be diagnosed with osteoporosis, and about half of them will require physician-supervised treatment following a fragility fracture [5–7]. A recent nationwide representative study in Korea has reported that the treatment rate for osteoporosis is only 12.8%, in the general population [4].

In an effort to prevent osteoporotic fractures, several educational intervention programs have been developed to improve osteoporosis diagnosis and treatment. However, most studies were focused on how educational intervention programs affected diagnosis and treatment of osteoporosis in hospitalized patients with hip fractures or other, bone-fragility fractures. Studies such as these (carried out a variety of intervention modalities including telephone call services, education, e-mail, and screening tests for osteoporosis) focused mainly on the improvement of diagnosis and treatment of osteoporosis [8–11]. The hypothesis of this study was the concept that an educational intervention program could improve osteoporosis knowledge, self-efficacy awareness and the dietary habits and customs of the senior population.

Therefore, the purpose of this prospective educational intervention study was to evaluate the relationship between in osteoporosis knowledge, osteoporosis self-efficacy, fall self-efficacy, physical exercise and dietary pattern of calcium and vitamin D intake, following increased awareness arising from increased osteoporosis education.

## Methods

The design and protocol of this study were approved by the Institutional Review Board of our hospital. All patients were informed that his or her medical data might be used in a scientific study. All patients provided consent.

### Participants

**Fig. 1:**
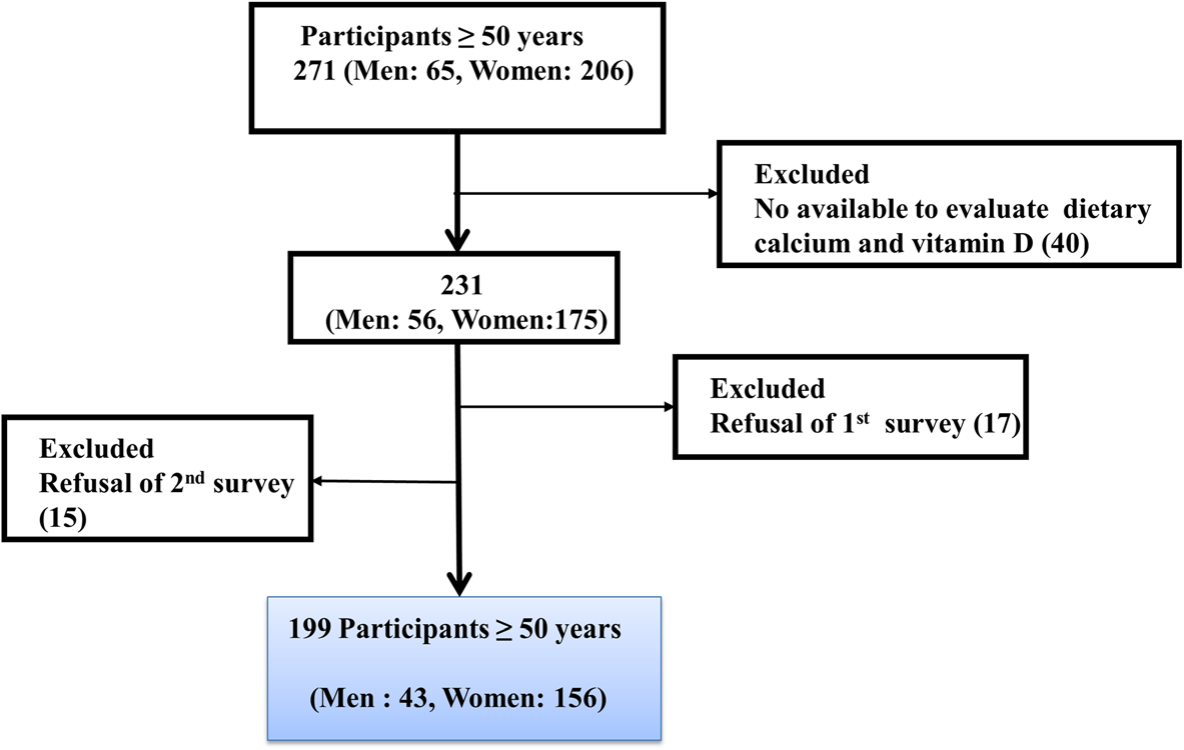
Flow chart for selection of study participants

From November 1, 2015 to August 31, 2016, 271 eligible candidates (individuals aged 50–95 years without cognitive dysfunction, able to walk, and without malignancy from 23 community centers were recruited through an announcement in the public office by two health care providers. Of these candidates, 40 (14.8%) were excluded due to an absence of available data necessary to evaluate their dietary calcium and vitamin D intake, 17 (6.3%) were excluded due to refusal of the first survey, and 15 (5.5%) were excluded due to refusal of the 2nd survey. After the aforementioned exclusions, a total of 199 participants (43 males and 156 females) were evaluated for the present study (Fig. 1). There were no differences between the characteristics of the 199 participants and those who were excluded.

### Educational intervention

The intervention involved an individualized education program, due to differing levels of education regarding several aspects of osteoporosis including actual comprehension of osteoporosis the disease, osteoporosis self-efficacy, falling self-efficacy risk factors and nutritional education stressing the importance of calcium and vitamin D intake. The participating patients were provided educational materials in print form (brochure developed by the Korean Society for Bone and Mineral Research). The exercise program consisted of stretching for the lower limbs, strength training for the lower limbs, balance training and impact training (all of which could be performed on a self-directed basis at home). All participants were educated in a formal program of therapeutic exercise, initially by a physical trainer and then they were strongly encouraged to keep on with the program, independently, afterwards. The researchers revisited the community centers three months after the initial visit. The consistency of compliance with the exercise program, at the final follow-up, was evaluated using a simple questionnaire. The follow-up reinforced the previously delivered educational messages and determined if any of the study endpoints were reached.

### Osteoporosis knowledge questionnaire

Each participant’s knowledge of osteoporosis was assessed using the facts as represented by an osteoporosis quiz (FOOQ) developed by Ailinger et al. [12–15]. These questions were based on the 2001 NIH Consensus Report on osteoporosis. This questionnaire is a validated and psychometrically sound instrument of minimal length that includes questions related to specific risk factors and specific self-care behaviors associated with an osteoporosis diagnosis. The FOOQ is worded at sixth-grade reading level. It has a content validity index of 0.87 and a Cronbach’s alpha of 0.76. The FOOQ consists of 20 true or false questions [16]. In addition to the ‘true’ and ‘false’ responses, there was a ‘don’t know’ response option. This third option allowed respondents a choice (for the precise purpose of reducing guessing so that a specific lack of knowledge, “don’t know response” and misinformation (incorrect response) could be identified and segregated from each other. In calculating the total raw score for the instrument, each item was assigned a score of “1” for a correct answer and a “0” if the answer was incorrect or ‘don’t know’. A total possible score for the FOOQ ranged from “0 to 20”, with higher scores indicating greater comprehension of osteoporosis the disease, and its ramifications.

### Osteoporosis self-efficacy questionnaire

Self-efficacy was measured using a modified ‘Osteoporosis Self-Efficacy Scale’ developed by Horan et al. [17]. To evaluate osteoporosis self-efficacy, questionnaire derived from research by Oh et al. [18]. They developed a new questionnaire from that created by Horan et al. [17] by converting it into a form on a scale of 5 points and it underwent verification process. The scale consists of 21 items with two subcategories, exercise (10 items) and calcium (11 items). Each of the 21 items was measured on a five-point scale (0 = not at all to 4 = extremely). Thus, the total score ranged from “0” to “84”, with higher scores corresponding to higher osteoporosis self-efficacy.

### Fall self-efficacy questionnaire

The fall self-efficacy questionnaire (Falls Efficacy Scale: FES-I) is a sixteen-item questionnaire developed to assess fall-related self-efficacy based on Falls Efficacy Scale (10 items) [17, 19]. It has six additional items including questions concerning the discharge of more difficult functional tasks and the social ramifications of falling [20]. In order to measure FES-I, questionnaire from research by Jang et al. [21] was used. The sixteen items of FES-I were rated according to “how concerned you are about the possibility of falling” using a visual analogue form anchoring between 0 representing ‘not at all confident’ (0) and 100 indicating ‘Very confident’. Thus, its total score ranged from 0 to 1600 points.

### Dietary calcium and vitamin D intake questionnaire

The dietary calcium and vitamin D intake questionnaire (Korean Calcium Assessment Tool: KCAT) was developed based on Calcium Calculator™ created by registered dietitians working for the BC Dairy Foundation in the 1980s [22]. The KCAT included seven food groups consisting of 24 categories, 45 food items containing calcium and vitamin D consumed frequently by Koreans. Food items with similar calcium content were put into one category. Average calcium and vitamin D intake was applied according to the Korean Standard Food Composition Tables (8th revision) the daily calcium and vitamin D intake from fluid according to recommended daily amounts of 700 mg. and 15 μg. in men and 800 mg. and 15 μg. in women, respectively. Frequency of servings was recorded as the number of servings per day, per week, or per month [23].

### Statistical analyses

In the first phase of statistical processing, all variables were assessed using the Kolmogorov-Smirnov test normality of distribution to establish variables’ parametric/nonparametric nature. Because the Kolmogorov-Smirnov test identified all the variables (except AUDIT) as non-parametric (also known as ordinal, descriptive statistics which included calculation of counts and frequencies).

We calculated the required study sample size using the model of Korean Fracture Risk Score [24], in which the mean risk of osteoporotic fracture in Ibansumg-Meon during sevenyears was 22%. Of risk factors, exercise could reduce 7% of fracture risk. Based on a power of 80%, significance level of 5%, and the estimated reducing rate of osteoporotic fracture of 7% in participants, 164 patients were found to be the optimal sample size. Expecting a drop-out rate of 20% during follow-up, 197 patients were included in the intervention study. Paired t-test was used for continual variables. *P*-values were reported two-sided, with *P* < 0.05 indicating statistical significance. Statistical analyses were performed using SPSS software version 19.0 (SPSS; Armonk, New York, USA).

## Results

**Fig. 2:**
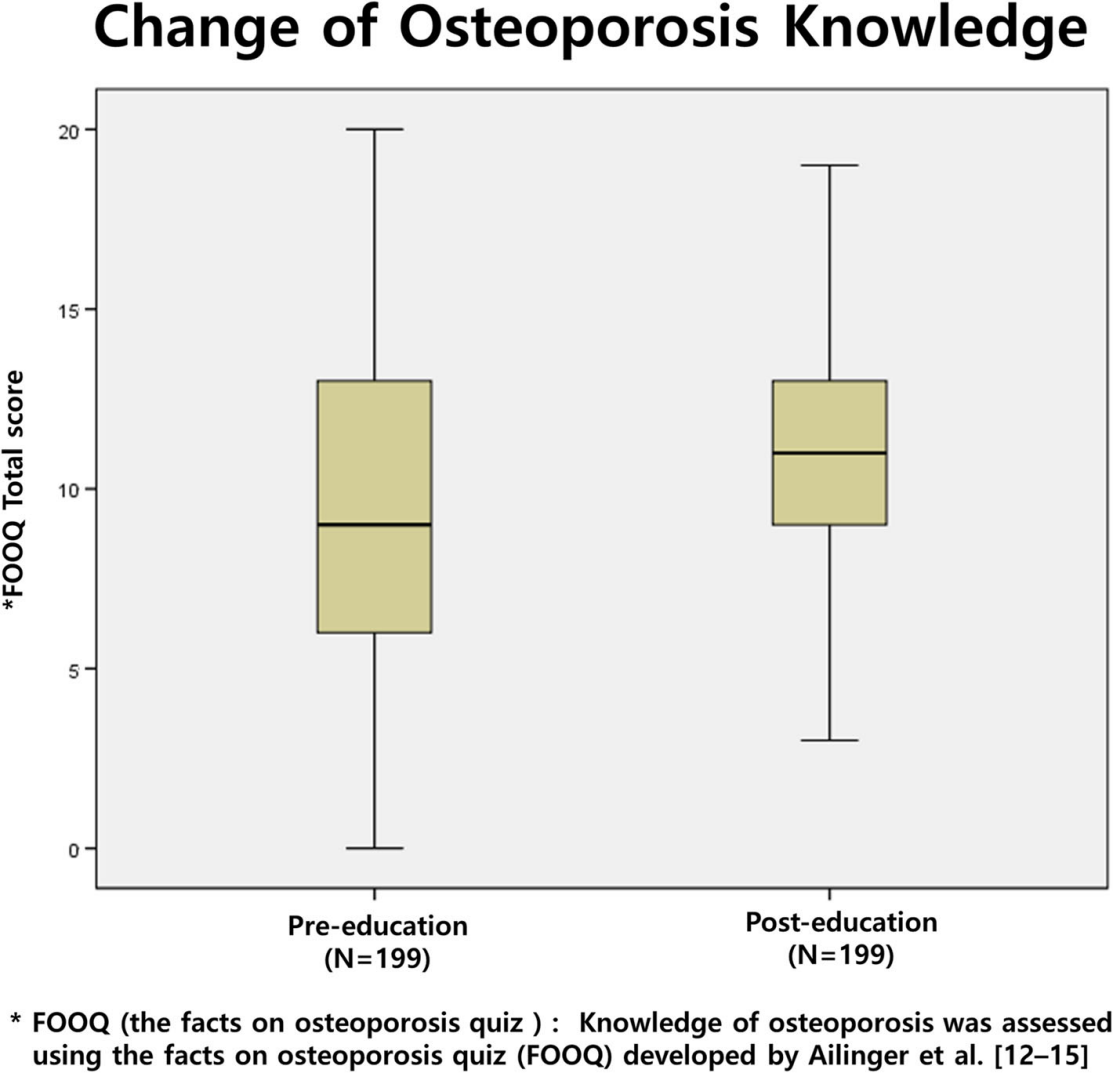
Change of Osteoporosis knowledge after the education program

**Fig. 3:**
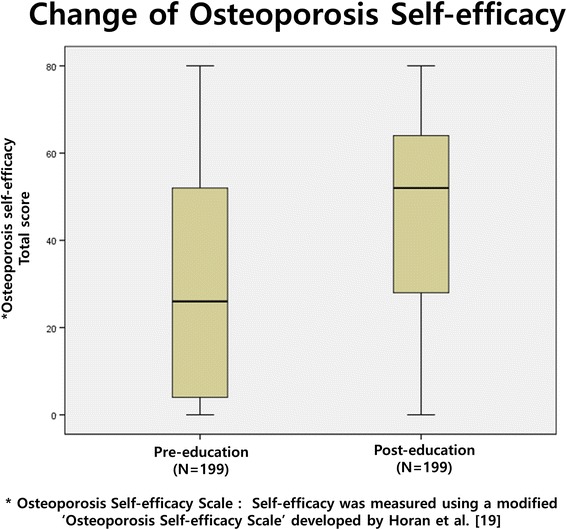
Change of Osteoporosis Self-Efficacy after the education program

Of the 271 potential participants, 199 (73.4%; 43 men and 156 women) completed both the education program and the second questionnaire. Of these participants, 159 (79.9%) were farmers; 162 (81.5%) graduated from elementary or had no formaleducation. The median age of these participants was 76.3 years (IQR 72–81.5, IQR = Interquartile Range). The median age was 74.9 years (IQR 67.8–81.0) for men and 76.6 for women. (IQR 72.0–82.0) Demographic characteristics of these 199 participants are summarized in Table 1. After education intervention, the osteoporosis knowledge score had increased from a median of 9.1 (IQR 6–13) at pre-education (baseline) to 11.2 (IQR 11–13) at post-education (*P* < 0.0001, Fig. 2). Of these 199 participants, 124 (62.3%) participants had improved osteoporosis knowledge scores while 60 (30.2%) had worse scores after the education intervention. The median of the osteoporosis self-efficacy score had improved (*P* < 0.0001) from 29.6 (IQR 4–52) to 46.1 (IQR 28–64) after the education intervention (Fig. 3).

**Table 1:** Demographic characteristics

Number	199
Men/Women	43/156
Age (Median) year	76.3 (IQR, 72.0–81.5)
Men	74.9 (IQR, 67.8–81.0)
Women	76.6 (IQR, 72.0–82.0)
BMI (Mean ± SD) (kg/m^2^)	22.4 ± 3.0 (range, 15.4–32.1)
Men	22.5 ± 3.0
Women	22.4 ± 3.0
Education level (school)	
Elementary	162 (81.5%)
Middle	19 (9.5%)
High	15 (7.5%)
University	3 (1.5%)
Occupations	
Farmer	159 (79.9%)
Housewife	15 (7.5%)
Employee	3 (1.5%)
Public officer	1 (0.5%)
Business	4 (2.0%)
No works	16 (8.0%)
Housemates (number)	
1	70 (35.2%)
2	107 (53.8%)
3–4	20 (10.1%)
More than 5	2 (0.9%)

*IQR* Interquartile range

**Fig. 4:**
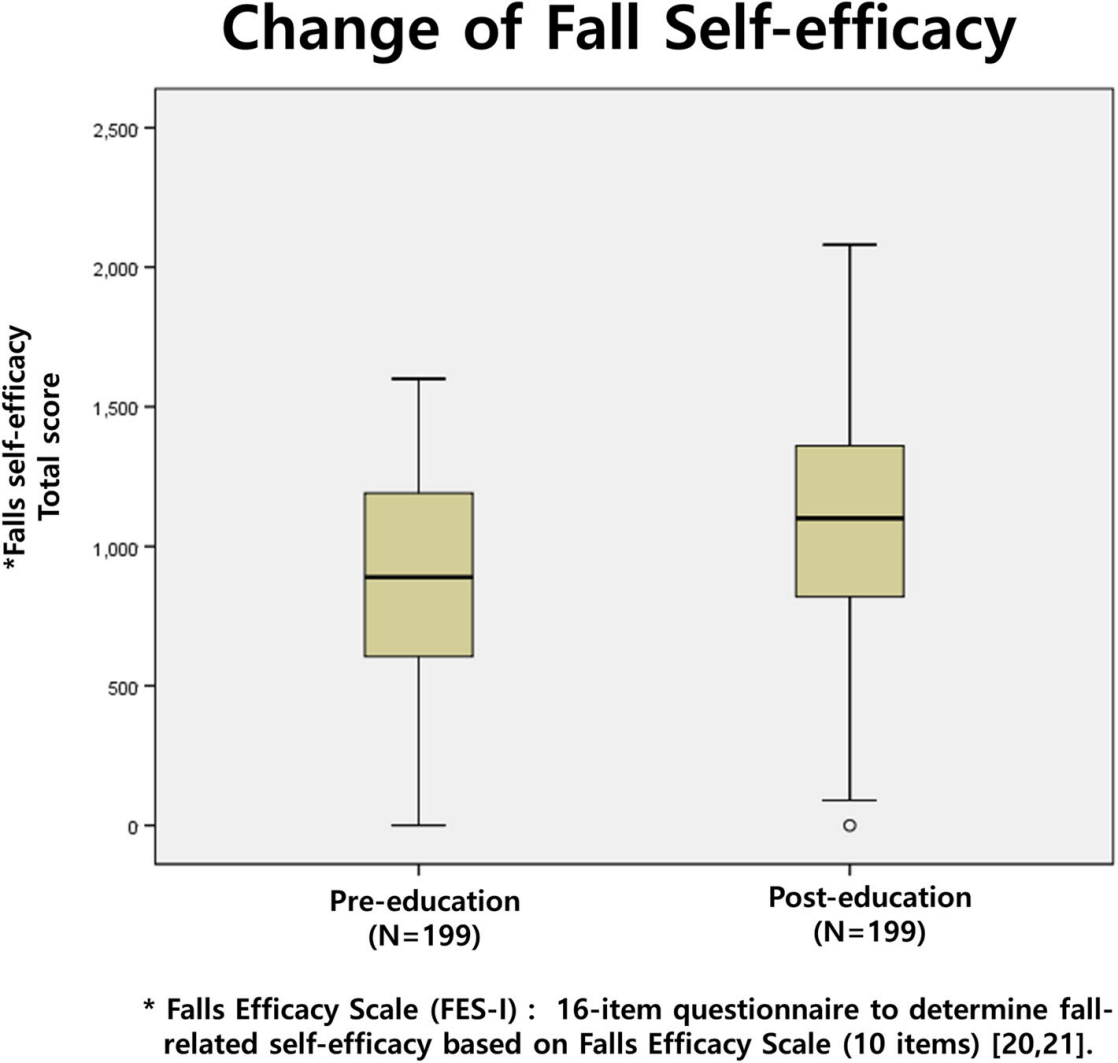
Change of Fall Self-Efficacy after the education program

To determine the exercise efficacy for obviating preventable falls, the median score of fall-related self-efficacy score was noted to have improved (*P* < 0.0001) from 893.0 (IQR 814.3–1360.0) to 1041.8 (IQR 603.8–1192.5) (Fig. 4).

Although fall-related self-efficacy was improved in 132 (66.3%) participants, it was unchanged or actually decreased in 67 (33.7%) of the participants at the time of the three-month follow-up evaluations.

After education regarding percentage of calcium and vitamin D intake below the recommended cut-offs, inadequate dietary calcium and vitamin D intake were decreased (*P* < 0.0001) from 89.4% (178/199) and 84.4% (168/199) to 79.9% (159/199) and 65.8% (131/199), respectively, at the time of the three-month follow-up. (*P* = 0.038, *P* = 0.017).

## Discussion

The objective of this prospective educational intervention study was to determine whether osteoporosis knowledge, osteoporosis self-efficacy, fall self-efficacy, and dietary calcium and vitamin D intake would improve after osteoporosis education and exercise programs.

The principle findings of this study served to demonstrate that when the osteoporosis knowledge score improved from 9.1 to 11.2, the osteoporosis self-efficacy score improved from 7.4 to 11.5, and the fall self-efficacy improved from 893 to 1041.8. In addition, patterns, habits and customs of dietary intake in the individual participants were adjusted to improve mean amounts of dietary calcium and vitamin D intake.

Interventional studies have consistently proven that osteoporosis knowledge, osteoporosis self-efficacy and fall self-efficacy levels all improved after the intervention [25, 26]. Evenson AL et al. [27] have observed the knowledge and awareness of material ramifications of a diagnosis of osteoporosis, health beliefs, self-efficacy, dietary calcium, and vitamin D intakes as measures of preventive behaviors with 153 young adults by using pre- and post- questionnaires including osteoporosis knowledge test, health belief scale, self-efficacy scale, and three-day food records. They reported that knowledge and self-efficacy of osteoporosis were improved after educational intervention. Olsen CF et al. [28] have performed a randomized clinical trial after exercise program and education on the fear of falling among 89 women with osteoporosis and documented history of vertebral fracture diagnosis. They found a significantly better result for the intervention group compared with the conservative group both at three months (*p* = 0.004) and twelve months (*p* < 0.001) follow-up. The size of the effect, at three months, was nominal (0.4). However, the effect was moderate (0.7) at twelve months. In this study, we also confirmed effectiveness of education and exercise intervention in community dwelling participants.

Although educational intervention for osteoporosis are frequently focused on BMD and anti-osteoporosis treatments [27, 29, 30], intervention studies regarding changes in life style such as dietary pattern are seldom reported.

Education programs including nutritional education may be effective in increasing dietary calcium and vitamin D intake of community-dwelling populations. Zhao, et al. [31] conducted a prospective randomized control study after nutritional education and dietary intervention for osteoporosis with 90 middle-age and elderly osteoporosis patients. After educational intervention, they found that daily intake of protein, vitamin, calcium, and dietary fiber of the intervention group were significantly higher than those of the control group. Lv and Brown [30] conducted a prospective educational intervention study for increasing intake of calcium rich foods.

After three months of follow-up, they found that the experimental group significantly increased calcium and vitamin D intake at post-test and such increase was maintained at the time of the follow-up. In this prospective study, we found that education can change food intake patterns, habits and customs, to favor and include food items with high content of calcium and vitamin D. After education intervention, dietary calcium intake increased from 446.8 mg. to 537.4 mg., and vitamin D intake increased from 8.3 μg. to 11.7 μg., consistent with previous findings revealed by previous studies. However, improvement of calcium and vitamin D intake after the intervention study was not sufficient to meet recommendations of calcium and vitamin D intake. These findings are generalized issues in the Korean population. According to the Korean National Health and Nutrition Examination Survey database, mean calcium intake and serum 25 (OH)D level were 485 mg./day and 48.1 nmol./L, respectively in Korean people [32].

Therefore, to improve calcium and vitamin D intake in community dwelling participants, nationwide countermeasures including calcium and vitamin D supplements may be both advisable and necessary.

This study has some limitations. First, the education level and old age of participants made it difficult for some to understand the effects of osteoporosis knowledge or follow the physical training.

Second, although all participants were provided exercise programs by a physical trainer in one-hour sessions three times weekly, it may not be sufficient to achieve the goal of intervention. Although we could not monitor during the whole period of exercise program, we confirmed the effects of the program without satiable recognition of participants’ compliance.

Third, there is no control group in this study and it may be difficult to deduce concrete conclusions about efficacy and impact. Despite these limitations, we hope our results serve as a basis for estimating the positive, long-term effects after intervention, such as increased awareness as to the importance of exercise and education, in other studies.

Fourth, this study could not measure prevalence of osteoporosis and fractures due to limitations of the study design. However, we can confirm improvement of osteoporosis knowledge and self-efficacy and these improvements may have resulted in preventable osteoporosis and related fractures. In addition, the three month study periods in this study could not able to confirm clinically significant effects such as reducing incidence of fall and/or fracture incidence. Gillespie et al. [33] performed systemic review to assess effect of interventional study such as education including knowledge of osteoporosis, exercise, and nutrient support and improvement awareness, exercise, and using 159 trials with 79,193 participants. They found that exercise programs aimed at reducing falls appear to, in turn, reduce the incidence of osteoporosis-related fractures. Fracture liaison service (which consists of education, screening test for osteoporosis, and pharmacological treatment) is proven to prevent second osteoporotic fractures [34, 35]. In fracture liaison service, patient’s education includes improving awareness, exercise, and calcium and vitamin D rich food intake is essential programs [34, 36]. Therefore, we think that improved awareness regarding importance of exercise, comprehension of osteoporosis as a pathology, and calcium- and vitamin D-rich food intake in community dwelling participants could induce positive changes in lifestyle, in turn serving to produce decreasing incidents of falls and/or other osteoporotic-related fractures.

However, to confirm a clinically significant effect, longer follow-up study is necessary to further demonstrate a cause-and-effect relationship between improvement of awareness and positive and therapeutic changes in life style, and reducing incidents of falling and consequential osteoporotic-related fractures in community dwellings which house an elderly population. Further research will be necessary to identify the relationship between change of self-efficacy and a diminution in fracture rates.

Finally, although calcium and vitamin D intake increased after intervention, the documented increases were insufficient vis-a-vis the recommended intake. In conclusion, follow-up study is felt to be both advisable and necessary to confirm the long-term clinical significance of effects after intervention.

## Conclusions

This prospective intervention study has served to demonstrate that education intervention could improve osteoporosis knowledge, osteoporosis self-efficacy, fall self-efficacy and serve to increase dietary calcium and vitamin D intake in community dwellings housing elderly populations.

## Abbreviations

FES: Fall efficacy scale
FOOQ: Facts on osteoporosis quiz
KCAT: Korean calcium assessment tool
